# Development of Controlled-Release Carbamide Peroxide Loaded Nanoemulgel for Tooth Bleaching: In Vitro and Ex Vivo Studies

**DOI:** 10.3390/ph14020132

**Published:** 2021-02-07

**Authors:** Siriporn Okonogi, Adchareeya Kaewpinta, Sakornrat Khongkhunthian, Pisaisit Chaijareenont

**Affiliations:** 1 Department of Pharmaceutical Sciences, Faculty of Pharmacy, Chiang Mai University, Chiang Mai 50200, Thailand; 2 Research Center of Pharmaceutical Nanotechnology, Chiang Mai University, Chiang Mai 50200, Thailand; sakornratk@hotmail.com (S.K.); yodent@hotmail.com (P.C.); 3 Interdisciplinary Program in Nanoscience and Nanotechnology, Faculty of Science, Chiang Mai University, Chiang Mai 50200, Thailand; akaewpinta@gmail.com; 4 Department of Restorative Dentistry and Periodontology, Faculty of Dentistry, Chiang Mai University, Chiang Mai 50200, Thailand; 5 Department of Prosthodontics, Faculty of Dentistry, Chiang Mai University, Chiang Mai 50200, Thailand

**Keywords:** carbamide peroxide, solid dispersion, nanoemulsion, nanoemulgel, controlled release, tooth bleaching efficacy, enamel microhardness

## Abstract

Burst release of carbamide peroxide (CP) from traditional hydrogels causes severe inflammation to periodontal tissues. The present study explores the development of a novel CP nanoemulgel (CP-NG), an oil-in-water nanoemulsion-based gel in which CP was loaded with a view to controlling CP release. CP solid dispersions were prepared, using white soft paraffin or polyvinylpyrrolidone-white soft paraffin mixture as a carrier, prior to formulating nanoemulsions. It was found that carrier type and the ratio of CP to carrier affected drug crystallinity. Nanoemulsions formulated from the optimized CP solid dispersions were used to prepare CP-NG. It was found that the ratio of drug to carrier in CP solid dispersions affected the particle size and zeta potential of the nanoemulsions as well as drug release behavior and tooth bleaching efficacy of CP-NG. Drug release from CP-NG followed a first-order kinetic reaction and the release mechanism was an anomalous transport. Drug release rate decreased with an increase in solid dispersion carriers. CP-NG obtained from the solid dispersion with a 1:1 ratio of CP to the polymer mixture is suitable for sustaining drug release with high tooth bleaching efficacy and without reduction of enamel microhardness. The developed CP-NG is a promising potential tooth bleaching formulation.

## 1. Introduction

Tooth discoloration is a dental problem that requires effective treatment because it affects physical appearance, beauty, and self-confidence. In adolescence, tooth discoloration can influence psychosocial development [1]. Moreover, the occurrence of tooth discoloration associated with poor oral hygiene is the main cause for the accumulation of pathogenic bacteria in the oral cavity leading to several severe dental infectious diseases such as gingivitis, periodontitis, and dental caries [2]. Many procedures are applied in order to treat tooth discoloration such as microabrasion, veneers, jacket crowns and tooth bleaching [3]. Among these, tooth bleaching is the most popular treatment because it is the simplest, least invasive, and least expensive for eliminating tooth color stains [4].

Tooth bleaching or tooth whitening agents using nowadays are mostly based on hydrogen peroxide. Carbamide peroxide (CP), one of the hydrogen peroxide precursors, is a potent oxidizing agent and has a high ability to produce peroxides that can diffuse through the organic matrix of the enamel and dentin and attack the long-chained colored molecules and split them into smaller, less colored, and more diffusible molecules. Moreover, CP can produce free radicals such as perhydroxyl anions and hydroxyl radicals which are very reactive [5]. The obtained free radicals can react with other molecules at the unsaturated bonds, resulting in disruption of electron conjugation and a change in the absorption energy of the organic molecules in tooth enamel. These mechanisms create a successful tooth bleaching action [6].

According to the American Dental Association, CP is categorized as an effective tooth bleaching agent that can be used during the day or at night time with a suitable dosage form [7]. Most of the bleaching products using nowadays are in a hydrogel dosage form which have hydrophilic gel bases made of various kinds of gelling agents such as carboxyvinyl polymers, alginates, carrageenan, and cellulose derivatives. However, the burst release of CP from these hydrogels leading to the severe inflammation of the periodontal soft tissue at the contact lesion is usually observed during the applications [8]. Although published studies tend to suggest that bleaching with low CP concentration from 10% to 16% is a relatively safe procedure [9], however, even at low concentration, the high amount of free radicals that are fast released from the bleaching products can diffuse through the dental tissue to the pulp chamber [10]. The free radicals can induce negative effects such as lipid peroxidation, fragmentation of proteins, and cell membrane injury [11]. Several studies have reported the disadvantages of the fast-release 10% CP gels, e.g., induction of inflammation in the dental pulp of premolars [12], a decrease in surface hardness [13], alteration of the surface roughness [14], and induction of enamel surface damage [15]. Moreover, the appearance of clinical side effects, e.g., tooth sensitivity [16] and gingival irritation [17] were also reported. In addition, carboxy polymethylene or carbopol which is commonly used as a gelling agent in CP gels also causes a decrease in enamel microhardness [18]. Therefore, CP gels need additional improvements. Rice possesses anti-inflammatory and antioxidant activities [19]. Our group previously reported that chemical modified rice can act as a suitable gelling agent for hydrogel formation [20]. The obtained rice gel base can be used as a mucosal drug delivery system [20,21]. The advantages of rice in these activities can provide a suitable hydrogel prepared from rice for oral application, particularly at the lesion where inflammation may occur. The gelling property of rice gel is dependent on rice variety [22]. Various varieties of rice were previously reported for the preparation of bleaching hydrogels containing CP. They showed different characteristics and properties such as rheological behavior, adhesion, viscosity, and drug release as well as bleaching efficacy according to the varieties of rice [23].

Nanoemulgel is one of these nanoformulations. It is usually composed of a hydrogel as the external phase of an oil-in-water (o/w) nanoemulsion (NE). The extremely small droplet size of NE can increase the efficacy and stability of the products during storage. Nanoemulgels possess a dramatically higher adhesion property and viscosity than NE [24]. With these properties, nanoemulgel receives high attention as a drug delivery system for topical administrations, e.g., dermatological, oral, and buccal applications [25]. Therefore, using a potential natural gelling agent to formulate a suitable control-release nanoemulgel is expected to improve CP tooth bleaching formulation with fewer side effects and high efficacy. The aim of the present study was to develop CP loaded nanoemulgel (CP-NG) using modified rice as a natural gelling agent and to reduce the burst release of CP by the controlled release formulation of o/w NE obtained by using a solid dispersion technique to entrap CP in the internal oily phase of NE. The influence of CP to carrier ratio in solid dispersion was investigated. The developed NE formulations were screened for permissible size and size distribution before incorporation with the gel bases. The rheological and adhesion properties, in vitro drug release behavior, ex vivo efficacy as well as the impact on tooth surface microhardness of the optimized nanoemulgels, were evaluated.

## 2. Results and Discussion

### 2.1. Preparation and Characterization of CP Loaded Solid Dispersions (CP-SD)

**Figure 1:**
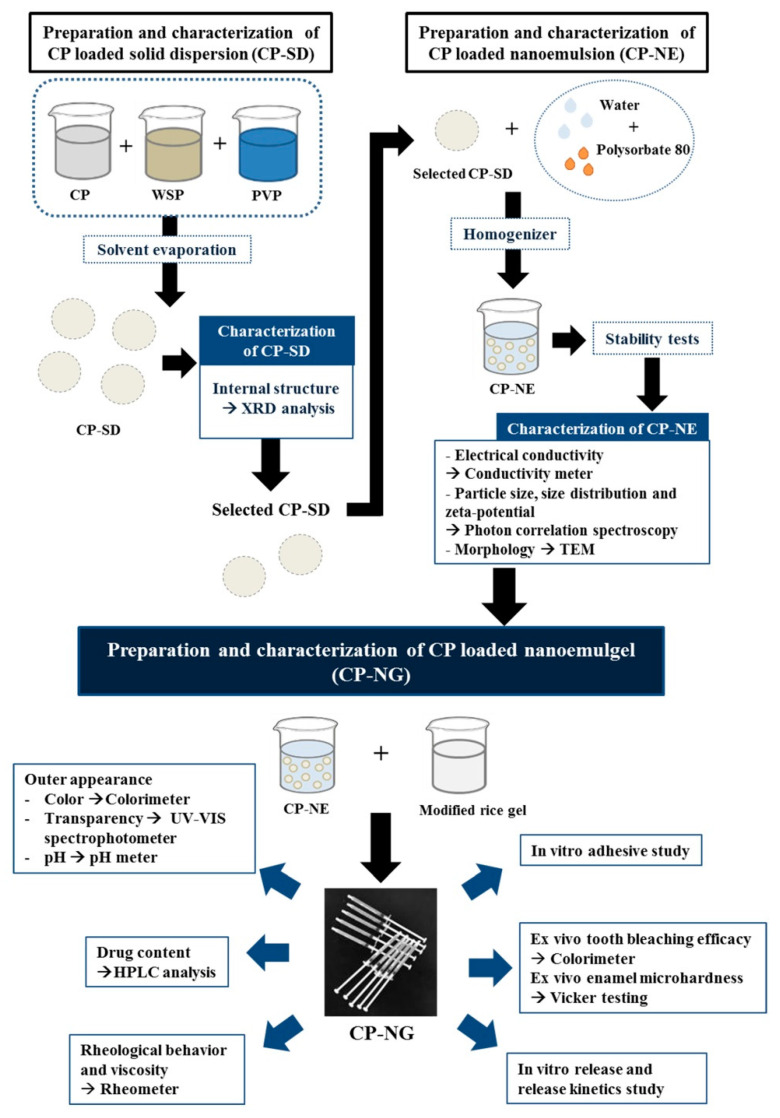
The preparation of CP-NG and analytical techniques used. (WSP = white soft paraffin, PVP = polyvinylpyrrolidone; XRD = X-ray diffraction; TEM = transmission electron microscope; HPLC = high-performance liquid chromatography).

**Figure 2:**
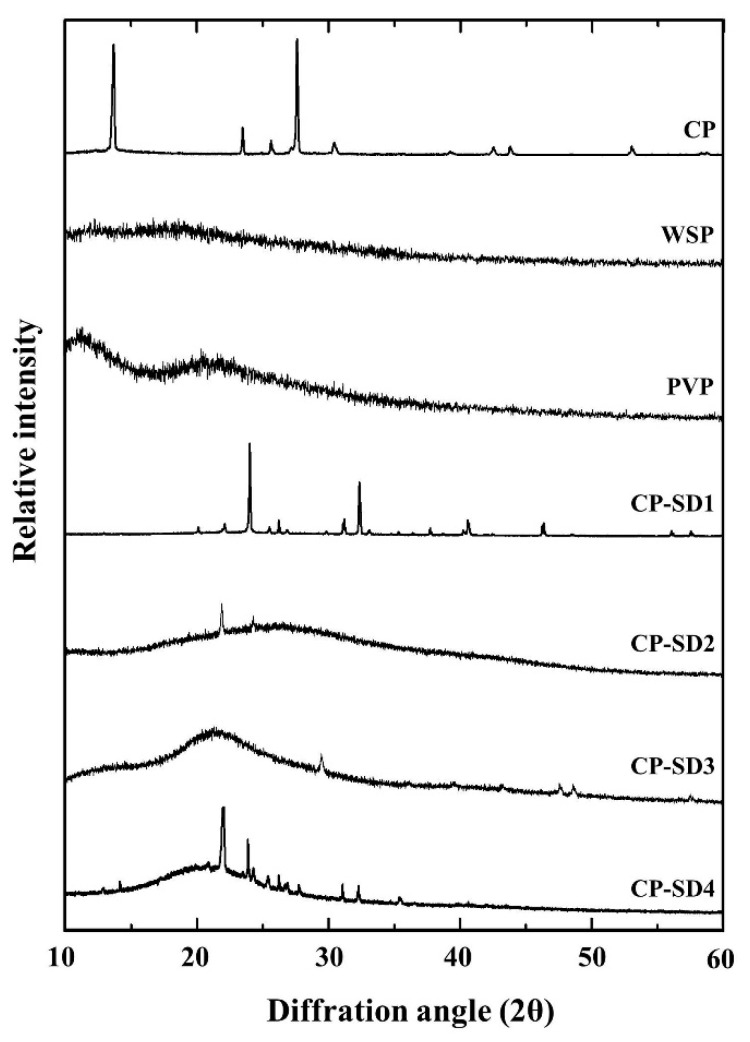
XRD patterns of CP-SD in comparison with different drug-carrier ratios and raw materials of CP and carriers.

All CP-NG formulations were prepared from CP-SD and CP-NE, respectively as summarized in Figure 1. Pharmaceutical solid dispersion is defined as the dispersion of a solid drug in an inert solid carrier. This system has an advantage for the solubilization of water-insoluble drugs [26] and stabilization of the unstable drugs against various decompositions such as hydrolysis and oxidation. CP is a strong oxidizing peroxide and easily to be decomposed. Binding the peroxide compound with an inert polymeric carrier in a solid dispersion is one of the potential methods for stabilizing the compound [27]. White soft paraffin (WSP) is a chemically inert hydrocarbon compound with different chain lengths. WSP can provide occlusion to the skin surface and increase the therapeutic effect of the active ingredients [28]. It can be used as an oily phase for the production of oil-in-water (o/w) emulsions and o/w creams. As WSP does not react with other active ingredients, therefore it is an excellent carrier material for many active ingredients and the obtained products can be relatively stable [29]. In the present study, WSP was firstly used as a carrier for preparing a mixture of 1:1 ratio of drug to carrier to formulate CP-SD1. The outer appearance of the obtained CP-SD1 was homogeneous white semi-solid. Characterization using X-ray diffraction (XRD) showed that the internal solid structure of CP was crystalline characteristics with the identical crystalline peaks at 2θ diffraction angles of 14°, 23°, 28°, and 32° whereas the halo pattern with an absence of peak was found in WSP diffractograms as shown in Figure 2. It was clearly observed that CP dispersed in the obtained CP-SD was also in crystalline form as two identical crystalline peaks of CP at 23° and 32° were observed. This can be attributed to a lack of miscibility and molecular interaction between hydrophilic CP and lipophilic WSP [30], which might be due to the practical immiscibility between hydrophilic CP and lipophilic WSP. To enhance the miscibility of these two compounds, the inert molecule having both hydrophilic and lipophilic parts should be added. Polyvinylpyrrolidone (PVP) is a biodegradable and biocompatible polymer approved by the US Food and Drug Administration and it is useful for the delivery of various drugs [31]. The chemical structure of PVP is composed of both hydrophilic and lipophilic moieties [32]. Moreover, PVP has been reported that it can stabilize hydrogen peroxide by binding with the peroxide to form a stable complex of PVP-peroxide [33]. Therefore, PVP was selected to mix with WSP to obtain a mixture of 1:1 ratio of PVP to WSP. This mixture was used as a carrier for CP-SD formation. The compositions of CP-SD with various amounts of CP and the carriers are shown in Table 1. After incorporating the PVP-WSP mixture with CP, the outer appearance of the obtained CP-SD was similar to that obtained from incorporation with only WSP. The halo patterns were found in the XRD patterns of CP-SD2 and CP-SD3. These results suggested that the dispersed CP in CP-SD2 and CP-SD3 was in amorphous form. The results indicated the advantages of PVP. As PVP possesses high glass transition property [34] and its molecular structure consists of hydrophilic-lipophilic moieties, these properties promoted PVP to inhibit the recrystallization of CP and improve the stability of the dispersed CP from aggregation in the solid dispersion. Many small crystalline peaks were observed in CP-SD4. Increasing carrier content as in CP-SD4 led to the presence of the drug in the crystalline form again. This result suggested that only the optimum carrier amount could retard the crystallization process of CP. From these results, the obtained CP-SD2 and CP-SD3 were selected for further study in NE preparation.

### 2.2. Preparation and Characterization of CP Loaded NE (CP-NE)

CP-NE1 and CP-NE2 were the NE formulations obtained from the selected CP-SD2 and CP-SD3, respectively. The compositions of CP-NE formulations and blank NE (BL-NE) are presented in Table 2.

The outer appearance of CP-NE1 and CP-NE2 was a homogeneous viscous liquid. The selected CP-SD was expected to be the oily internal phase dispersed as extremely small droplets throughout the aqueous external phase of the obtained o/w NE. To confirm the o/w type of NE, the electrical conductivity of the formulations was measured since the systems with high electrical conductivity represent the o/w type while the low or no electrical conductivity represents w/o NE [35]. The results as shown in Table 3 demonstrates high values of electrical conductivity of both CP-NE1 and CP-NE2 indicating that both systems are o/w NE. CP-NE2 showed a higher conductivity than CP-NE1. This may be due to the water content, as CP-NE2 contained a higher amount of water than CP-NE1. The o/w type of NE can be confirmed by diluting the samples with an equal volume of water. The aqueous external phase of the o/w type can be well miscible with the added water whereas the oily external phase of the w/o type cannot. From this experiment, it was found that both CP-NE1 and CP-NE2 could be well diluted with water (data not shown), confirming that both nanoemulsions are of o/w type. 

After subjecting to the stress condition of cooling-heating cycles, both CP-NE formulations showed high stability without any change in visual appearance, drug precipitation, phase separation, or color change. Therefore, both systems were selected for further investigation in particle size, size distribution, and zeta-potential. The results as presented in Table 3 demonstrate that the size of CP-NE1 and CP-NE2 was significantly larger than that of BL-NE1 and BL-NE2, respectively, indicating that the loaded CP caused an enlargement of the droplets which were the internal phase of the NE. Moreover, the particle size of CP-NE2 was significantly larger than that of CP-NE1. This result suggested that the important role of the ratio of drug to a carrier in solid dispersion. The polydispersity index (PdI) values of all NE formulations were in the range of approximately 0.2–0.3. These PdI values are acceptable for lipid-based carriers drug delivery systems and indicating the homogeneity of the particle size [36]. The zeta potential of all CP-NE formulations was negative having a value forward to nearer zero than that of their respectively BL-NE formulations. These results indicated that CP could significantly affect the zeta potential of the NE. Comparing the zeta potential of both CP-NE formulations, CP-NE2 showed significantly higher negative values than CP-NE1. This result suggested that the amount of carrier in solid dispersion might affect the zeta potential of the NE. The molecular structure of PVP possesses negative charges according to the carbonyl group and nitrogen atom of the pyrrolidone ring [37]. These negative-charge groups were considered to play an important role in increasing the negative zeta potential value. Therefore, the higher negative zeta potential value of CP-NE2 might be due to the higher content of PVP in the system.

**Figure 3:**
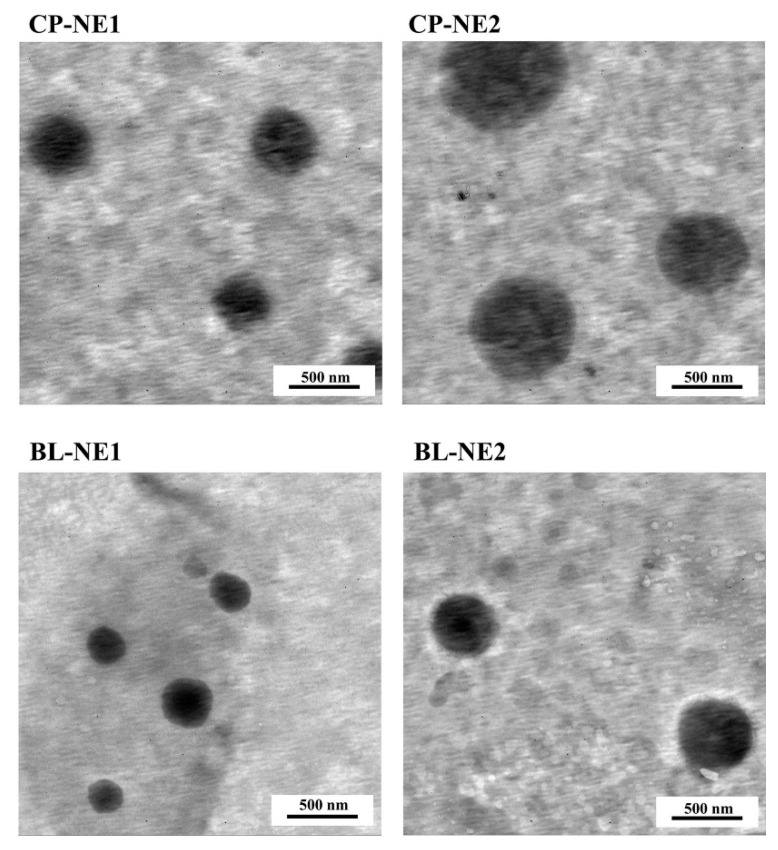
TEM images of CP-NE compared to BL-NE at magnification of 10,000×.

The obtained NE formulations were subjected to a transmission electron microscope (TEM) to investigate the particle morphology and distribution of the droplets of the internal phase. The morphology of CP-NE formulations as shown in Figure 3 reveals a spherical shape in the nanometer size range of the internal phase droplets of the NE. All droplet particles were distributed with non-aggregation suggesting high stability of the formulations against Oswald ripening due to globular collapse [38]. Adhesion property is one of the key criteria for bleaching products, as the products should have sufficient adhesion to be at the tooth surface or application area throughout the desired duration of action. CP-NE1 and CP-NE2 were compared with a commercial gel containing the same amount of CP on this property. It was found that the adhesion properties of CP-NE1 and CP-NE2 were approximately 10 times less than that of the commercial gel as shown in Table 4. These results suggested that CP-NE1 and CP-NE2 needed further development to have a higher adhesion property.

### 2.3. Preparation of CP-NG

Nanoemulgel combines the advantages of NE and hydrogel leading to the optimum control of drug release. Nanoemulgel possesses gel characteristics that can improve the disadvantages of NE for local application, e.g., it can enhance the adhesion for oral cavity application [25]. Nanoemulgel can potentially act as a drug reservoir, influencing the release of drugs from the inner phase to the outer phase and has contributed to the efficient, safe, and successful delivery of many therapeutic agents [39]. Therefore, nanoemulgel has been considered as a suitable delivery device for CP in the present study. After incorporating the selected CP-NE1 and CP-NE2 with modified rice gel, two nanoemulgels having 10% CP, CP-NG1, and CP-NG2 respectively were obtained.

### 2.4. Characterization of CP-NG

CP-NG1 and CP-NG2 were characterized in comparison with CP-RG (10% CP in rice gel), positive control (CP-CG), and the gel base as follows.

#### 2.4.1. Outer Appearance, Drug Content, and pH

From visual observation, CP-NG1 and CP-NG2 had a translucent viscous with a glossy appearance and a smooth and homogeneous texture without phase separation or drug precipitation. Subsequently, the gel formulations were analyzed for their outer appearance by measurement of color and transparency. Colorimetric measurement was done according to the Commission International de l’Eclairage (International Commission on Illumination: CIE) L*a*b* color parameters (CIE L*a*b* system). This measurement reflects three color parameters: L*, a*, and b*. The parameter L* refers to the lightness and ranges from black to white (L = 0–100). The parameter a* refers to red-green color, a positive a* refers to red and a negative a* refers to green. The parameter b* refers to yellow-blue color, a positive b* indicates yellow, and a negative b* indicates blue. The differences (∆) in L*, a*, and b* parameters between the gel and the white background was presented in Table 5. The ∆L* value of both CP-NG was higher than hydrogel, i.e., CP-RG, CP-CG, and gel base. Therefore, the incorporation of CP-NE to the gel base caused the lightness values to increase significantly. Regarding ∆a* and ∆b* parameters, CP-NG exhibited lower values compared to the hydrogel. CP-NG was slightly blue green in color as the negative of a* and b* values whereas CP-RG and gel base was slightly yellow as the positive of b* values. The color difference (∆E) values were calculated using Equation (1):

(1) $$∆E\;=\;{\left[{\left(∆L*\right)}^{2}\;+\;{\left(∆a*\right)}^{2}\;+\;{\left(∆b*\right)}^{2}\right]}^{1/2}.$$

The ∆E values showed the degree of color difference for optical sensations. For ∆E less than 1, the color differences could not be noticeable to the human eye, ∆E in range of 1–3, the minor color differences could be noticeable to the human eye, and ∆E more than 3, the color differences could be noticeable to the human eye [40]. The ∆E values of both CP-NG was higher than 2 therefore the difference in color compared to hydrogel could be observable. The incorporation of CP to gel base did not change the color of gel base as the values of color parameter of CP-RG and gel base were similar, their color were almost colorless as the ∆E values was close to 1. The transparency of the gel formulations was analyzed in term of transmittance (%). As showed in Table 5, the transmittance of CP-NG was lower than CP-RG, CP-CG, and gel base. Comparing between both CP-NG, as the particle size of CP-NE increased, the transmittance tend to decrease. Therefore, the transparency of CP-NG is in direct proportion with the quantity of CP-SD carriers in NE formulations. For CP-RG, the incorporation of CP to gel base slightly increased transparency of the gel.

Measurement of drug content is important to ensure the presence of CP in the formulations, as well as drug uniform distribution. As presented in Table 6, the drug content in all formulations was equal to the amount of original added. This result indicated that the prepared CP-NG formulations had a uniform drug distribution and confirmed that the drug degradation did not occur during the process of nanoemulgel production. 

The pH of CP-NG1 and CP-NG2 was close to that of gel base which was nearly neutral, whereas that of CP-RG was slightly acid. Acidic or alkaline pH is potentially harmful to the enamel and dentin and soft tissues in the oral cavity. To reduce these side effects to the oral cavity, the pH of the formulation should be as close to neutral as possible [41,42]. The obtained CP-NG formulations had a comparable buccal pH, indicating that the developed formulations might have no or less irritation to the oral cavity after application.

#### 2.4.2. Rheological Behavior and Viscosity

**Figure 4:**
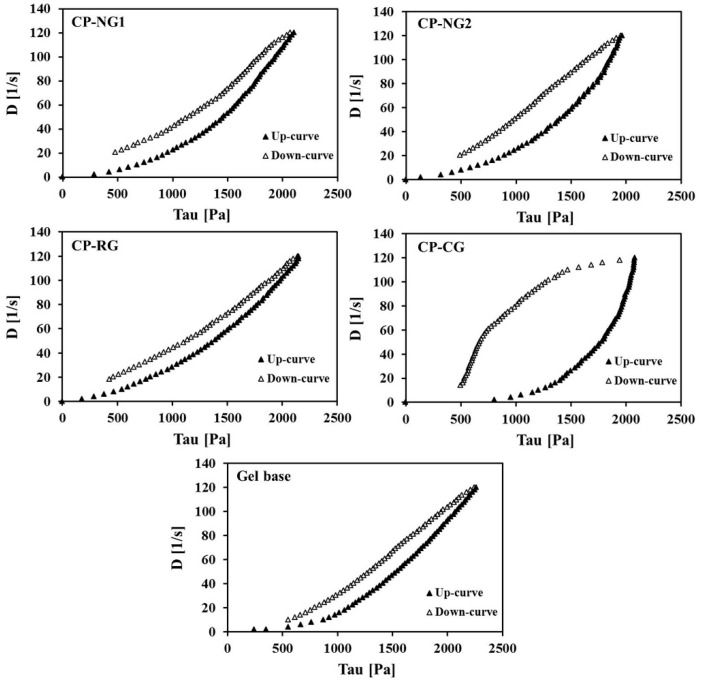
Rheograms of CP formulations and the gel base.

Rheology is a significant parameter for evaluation of the nanoemulgels especially for topical application. The microstructure and flow behavior information of topical nanoemulgel formulations needs to be evaluated for drug diffusion and compatibility. The relationship between the stress and the shear rate on the samples was measured to study flow behavior and to determine the viscosity [43]. The rheograms of the samples as presented in Figure 4 reveal that the rheological behavior of all formulations was non-Newtonian flow as the stress-strain relationship was not linear. All gel formulations showed pseudoplastic flow indicating an immediate flow after receiving stress. The rheograms of both CP-NG, CP-RG, and CP-CG displayed thixotropic characteristics as indicated by the differences observed in ascending and descending curves of the rheograms. The results suggested that incorporation of CP-NE had no effect on the rheological behavior of the rice gel base. Our previous study reported that the rice gel base exhibited pseudoplastic flow with thixotropy [23]. CP-CG also showed pseudoplastic flow with thixotropy. This behavior was found in carboxy polymethylene gel which is a synthetic polymer that usually used in tooth bleaching commercial gel [44]. Thixotropy is a reversible variation of viscosity with time and demonstrated by the presence of a hysteresis area between ascending and descending curves of the rheograms [43]. Pseudoplastic flow behavior with thixotropy is a desirable property for the semisolid dosage forms for topical application, as it could facilitate the application of the product on the surface [45]. 

The viscosity of the test formulations is shown in Table 6. Gel base exhibited significantly higher viscosity than all CP containing gels. The viscosity of CP-NG1, CP-RG, and CP-CG was similar to each other and significantly higher than that of CP-NG2 (*p* < 0.05). Considering the compositions used in CP-NG1 and CP-NG2, there was a difference in the quantity of gel base and water. The lower viscosity of CP-NG2 than CP-NG1 might be due to the less amount of gel base and higher amount of water used in CP-NG2 than that used in CP-NG1.

#### 2.4.3. Adhesion Property

Adequate adhesion is a primary requisite for the formulations to successfully deliver drugs for topical administration. High adhesion property can provide the formulation to place on the application area throughout the desired duration of action. In case of tooth bleaching activity, the formulations should have high adhesion properties to stay on the tooth surface. The results as shown in Table 6 indicated that both CP-NG1 and CP-NG2 possessed significantly (*p* < 0.05) higher adhesion strength than other CP formulations. It was noticed that although CP-NG2 had a slightly lower viscosity as compared to others, this formulation exhibited good adhesion property. These results confirmed the enhancing adhesion property of the developed nanoemulgel formulations.

### 2.5. In Vitro Drug Release and Release Kinetics

**Figure 5:**
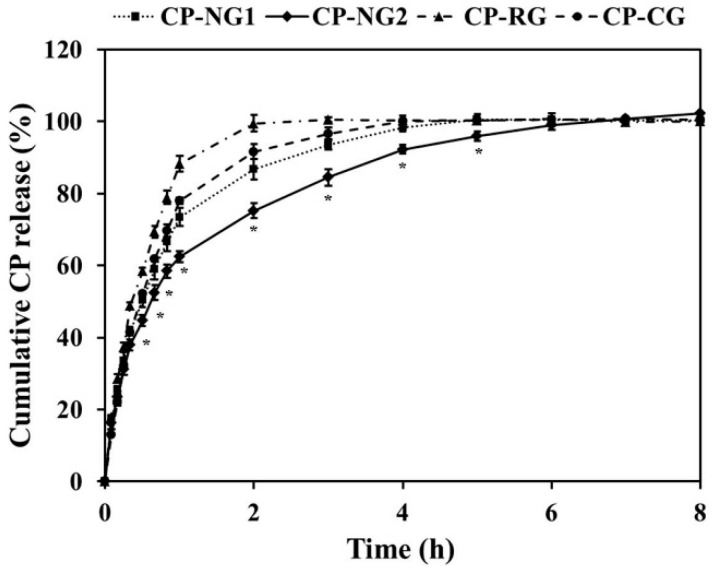
In vitro cumulative CP release from the gel formulations. * Statistical significance from the comparison of CP-NG2 to the other gel formulations at various time intervals (*p* < 0.05).

The ability of nanoemulgel formulations to deliver CP was examined by determining the released CP. The results as shown in Figure 5 showed that the cumulative percentage release of CP from all CP formulations at different sampling intervals was different during the first 5 h of the releasing period. Considering the developed CP-NG formulations, the releasing of CP from CP-NG2 was significantly slower than that from CP-NG1 (*p* < 0.05). However, at 6 h, the cumulative drug release of CP-NG2 reached 99.86 ± 1.43%, not significantly different from CP-NG1 and the other formulations, and after 6 h the cumulative drug release of CP-NG2 was completely the same as the other formulations. Considering the release manner, the hydrogel formulation, CP-RG, and the commercial gel product, CP-CG showed significantly faster drug release (*p* < 0.05) during the first 5 h than both developed slow-release CP-NG1 and CP-NG2 nanoemulgels. Drug releasing from hydrogel formulations normally occurs through the hydrophilic gel barrier, and the release rate is controlled by polymer swelling, polymer degradation, and drug diffusion [46]. The sustained drug release profile at the first 5 h of the developed nanoemulgel formulations can be mainly attributed to the polymer composition which is a carrier in CP-SD. The drug release from carriers is influenced by several factors including the composition, ratio, physical, and chemical interactions between the drug and the carriers [47]. CP-NG2 contained a high amount of carriers than CP-NG1, therefore this formulation demonstrated a slower CP release than CP-NG1. 

To investigate the release kinetics of CP from the preparations, a mathematical model such as a zero-order kinetic reaction such as Equation (2) or a first-order kinetic reaction as Equation (3) was applied:

(2) $${Q\;=\;k}_{0}t,$$

(3) $${\mathrm{Log}\;Q\;=\;\mathrm{Log}\;Q}_{0}\;-\;\frac{k_{1}t}{2.303},$$

where Q_0_ is an initial amount of drug in the formulations, Q is an amount of drug released at time t, and k_0_ and k_1_ are the rate constants of the zero-order and the first-order kinetics, respectively. The release data of the test formulations were fit to the mathematical models. The kinetic parameters could be estimated from the graphical plots (Figure S1) as shown in Table 7. The correlation (r^2^) values from a linear regression analysis of these mathematical models were compared, as the most appropriate kinetic model should show the highest correlation that the r^2^ value is equal to or near 1.0. It was clear from the r^2^ values that CP release from both developed nanoemulgels (CP-NG1 and CP-NG2) and the hydrogel, CP-RG as well as the commercial product, CP-CG followed the first-order kinetics that the drug release rate is concentration-dependent. These results confirmed that CP-RG possessed the highest drug release rate. Both CP-NG formulations showed significantly less k_1_ values than CP-RG and CP-CG, respectively. The sustained release rates are associated with the entrapment of CP in the nanoemulgel formulations. Comparing between CP-NG1 and CP-NG2, the k value of CP-NG1 was higher than that of CP-NG2. Considering the compositions of the CP-NG formulations, the slow release rate seemed to be in accordance with the increase of WSP-PVP in CP-SD. This result suggested that the composition of CP-SD influences the release rate of CP-NG. 

Higuchi and Korsmeyer-Peppas models were applied For the study of the release mechanism. The Higuchi model is the most widely used controlled release model from matrix systems. According to this model, the main mechanism of drug release is a diffusion process based on Fick’s law or Fickian diffusion as shown in Equation (4) [48]:

(4) $${Q\;=\;k}_{H}t^{1/2},$$

where k_H_ is the rate constant of Higuchi. 

Korsmeyer-Peppas model, as shown in Equation (5), is a useful mathematical model to study the drug release from hydrogel matrices when the release mechanism is not known or when more than one type of drug release phenomenon is involved [49]:

(5) $$M_{t}/M_{\infty }{=\;k}_{\mathrm{KP}}t^{n},$$

where M_t_/M_∞_ is a fraction of drug released at time t, k_KP_ is the drug release rate constant, and n is the diffusional exponent. The drug release mechanisms have been classified into three mechanisms based on n values: Fickian diffusion mechanism for n ≤ 0.5, non-Fickian release mechanism or anomalous transport for 0.5 < n < 1.0, and case-II transport mechanism for n ≥ 1.0 [50]. The first 60% of drug release data were analyzed by the Korsmeyer-Peppas equation. 

From the result of the kinetics study as seen in Table 7 (Figure S1), The Korsmeyer-Peppas model showed a higher value of r^2^ for all CP gel formulations. Therefore, the mechanism of CP release can be predicated via using this model. The obtained n values were found in a range of 0.52–0.71. This result suggested that the CP release mechanism from all CP gel formulations was an anomalous transport. This mechanism is correlated to a combination of drug diffusion and polymer relaxation [51]. 

### 2.6. Ex Vivo Bleaching Efficacy

**Figure 6:**
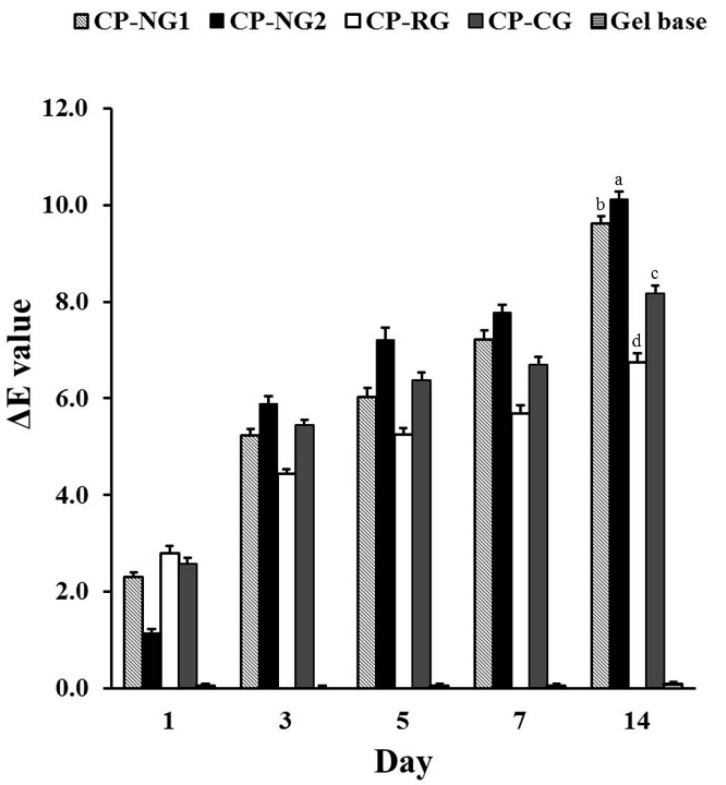
Ex vivo tooth bleaching efficacy of CP-NG in comparison to CP-RG, CP-CG, and gel base. Different lowercase letter implied the statistically different (*p* < 0.05).

The CIEL*a*b* system commonly uses in dental bleaching research. This system provides a useful tool for quantifying the color properties of teeth [52]. In the present study, the color changing was expressed in ∆E under CIEL*a*b* system. The results as presented in Figure 6 exhibited that all treatment groups significantly exhibited the tooth bleaching efficacy compare to the negative control (*p* < 0.05). ∆E values ranging from 4 to 8 were obtained from all treatment groups. It has been reported that ∆E values of at least 3.3 are visually perceptible [53]. Therefore, all of the treatment groups were considered to be effective for tooth bleaching. The results also showed that the CP-NG2 group possessed the significantly highest ∆E value (*p* < 0.05) followed by CP-NG1, CP-CG, and CP-RG, respectively. The results suggested that the nanoemulgel could induce a higher bleaching effect than a simple hydrogel. This idea would be supported by the fact that after the bleaching procedure completed, ∆E values of CP-NG were superior compare to other formulations. It was noticed that ∆E value of CP-RG was the highest after the first day of bleaching. However, after a complete bleaching procedure of 14 days, ∆E value of CP-RG was the lowest. A possible explanation for this result could be that a rapid decomposition of CP in the hydrogel may occur and lead to reduce in CP activity. 

Nanoemulgel formulation was previously reported that its ability to enhancing drug efficacy was along with prolonged residence time and reach greater surface area [54]. CP-NG2 exhibited high retention time of the drug on the tooth surface with sustained drug releasing and high adhesion. Moreover, as mentioned above, CP-NG had a higher pH than other CP gel formulations. From the literature, the bleaching effect of peroxide is accelerated by various factors such as higher temperature, catalyst, and higher pH [55]. Moreover, it was reported that the bleaching product of higher pH showed a greater bleaching effect [56]. This finding indicated that CP-NG formulations had high tooth bleaching efficacy due to the enhancement in CP activity as compared to a simple CP dispersion in the hydrogel base. In addition, comparing between both CP-NG formulations, CP-NG2 presented higher efficacy than CP-NG1. This result may be considered that CP-NG2 was the formulation that had a high amount of solid dispersion carriers, resulting in a stabilized nanoemulsion with a high negative zeta potential value, therefore CP-NG2 might have high product stability and the ability to protect CP from degradation during storage.

### 2.7. Effect on Enamel Microhardness

**Figure 7:**
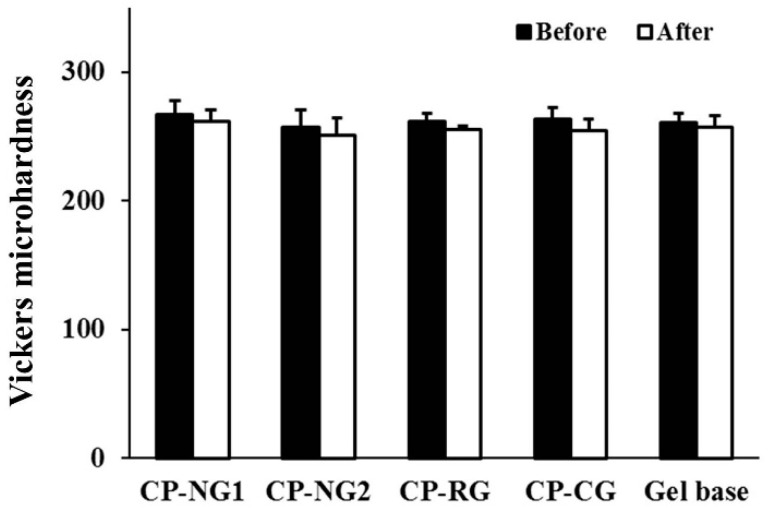
Values of the enamel microhardness, measured as Vickers hardness number, before and after bleaching by CP gel formulations and gel base.

Safety concerns of the tooth bleaching products are initially raised with the rapid growth of bleaching successfully, therefore, the effect of CP-NG on enamel microhardness was studied using Vickers’s hardness test. The values of enamel microhardness before and after the bleaching procedure are shown in Figure 7. After 14 days of bleaching procedure, the enamel microhardness values slightly reduced when compared to baseline. However, no statistically significant reduction of enamel microhardness values was found. Comparing the difference between the treatment groups and the negative control group, the results were also found to be insignificant. 

It is controversial in the literature whether bleaching gel has an effect on enamel microhardness. Previous studies reported a decrease in enamel microhardness after using 10% CP solutions [57] and 10% CP hydrogel [14]. In contrast, other studies reported no change of enamel microhardness of using 10% CP hydrogel [58,59]. Different results possibly due to the variations in methodologies such as the duration of bleaching, the amount of testing gel, and the setting of the controlled environment. 

Our results confirmed that using CP-NG with a treatment time of 8 h per day for a period of 14 days did not have a negative effect on enamel microhardness. One possible explanation is due to the pH of CP gel formulations. Generally, the reduction in enamel hardness is associated with pH values. An acidic pH leads to a superficial demineralization of the enamel, which can cause the dissolution of the tooth enamel and dental erosion, can occur [60]. The critical pH for enamel demineralization is approximately 5.5 [61]. From our investigations, none of the testing gel had a pH lower than 5.5, therefore it will less promote enamel demineralization. Moreover, it was noticed that CP-NG had a neutral pH than other CP gel formulations. Therefore, CP-NG would possibly reduce the risk of demineralization and ultimately minimizing the chance of negative effects for long-term treatment. In addition, a previous study reported that the gelling agent such as carboxy polymethylene and inactive ingredients such as glycerin, which are commonly used in commercial bleaching products, caused a decrease in enamel microhardness [18,62]. In our study, the enamel microhardness value in the gel base group was no difference from the baseline. Therefore, using the modified rice as a gelling agent in tooth bleaching gel product is considered safe.

Thus, it can be concluded that CP-NG effectively controlled CP release and enhance tooth bleaching efficacy without a negative effect on enamel microhardness. Further clinical evaluations are required to confirm the efficacy of CP-NG for human use. Patient satisfaction and side effects such as tooth sensitivity and gingival irritation in human subjects also need to investigate in future studies.

## 3. Materials and Methods

### 3.1. Materials

CP, triphenylphosphine, silver nitrate, monochloroacetic acid, PVP, and polysorbate 80 were obtained from Sigma Chemical Co. (St. Louis, MO, USA). Dichloromethane, absolute ethanol, methanol, glacial acetic acid, and WSP were from RCI Lab-scan Co., Ltd. (Bangkok, Thailand). CP-CG was from Ultradent Product Inc. (Salt Lake City, UT, USA). Rice seeds of Thai rice, variety Saohai, were obtained from the local market (Chiang Mai, Thailand). All other chemicals and solvents were of AR grade or the highest grade available.

### 3.2. Preparation and Characterization of CP-SD

CP-SD were prepared by solvent evaporation technique. WSP and mixtures of WSP and PVP were used as carriers. CP and the carrier solutions were prepared. Absolute ethanol was used as a solvent for CP and PVP whereas dichloromethane was for WSP. The prepared drug-carrier solutions were subjected to a rotary evaporator (EYELA N-1000, Tokyo, Japan) at a controlled temperature of 40 ± 5 °C to remove the solvents. The obtained CP-SD were characterized by means of XRD using a Siemens D-500 X-ray diffractometer with Cu Kα radiation at a voltage of 30 kV and 15 mA to investigate the internal solid structure of CP dispersed in the solid dispersions. The samples were loaded onto the sample holder of the diffractometer and scanned over a scanning rate of 12°/min with a Bragg angle (2θ) range of 10°–60°.

### 3.3. Preparation and Characterization of CP-NE

CP-NE were prepared from the optimum CP-SD. Polysorbate 80 and water was mixed using a vortex mixer. The obtained solution was added to the selected CP-SD at 40 °C with vigorous mixing. Subsequently, the mixture was subjected to a high-speed homogenizer (T25 digital Ultra-Turrax, IKA, Staufen, Germany) and stirred at 10,000 rpm for 5 min at 40 °C. For comparative purposes, blank BL-NE were prepared using the same procedure as CP-NE but the solid dispersion without CP was used instead of CP-SD. The compositions of CP-NE and BL-NE formulations are presented in Table 2. 

All NE formulations were subjected to stability tests prior to characterization. They were stored in six cooling-heating cycles between 4 °C and 45 °C by storing at each temperature for 24 h. The NE that did not show any phase separation, creaming, or precipitation after the test were selected for further study on characterization by determining electrical conductivity, particle size, size distribution, zeta-potential, and investigation of morphology.

The conductivity meter (D-24 Horiba, Kyoto, Japan) was used for the characterization of electrical conductivity. Before the measurement, the apparatus was calibrated using 1.41 mS/cm and 12.9 mS/cm conductivity standard solutions (LAQUAtwin, Horiba). The electrode (9625-10D, 3-in-1 Electrodes, Horiba, Kyoto, Japan) was inserted into the samples and the electrical conductivity measurement was carried out in triplicate at 25 °C.

The average size, size distribution, and zeta potential of the selected NE were determined by photon correlation spectroscopy using a Zetasizer Nano ZS-90 (Malvern Instruments Ltd., Malvern, UK). Prior to the measurements, the samples were diluted with milli-Q water (1:100, *v/v*) and then the dilution was filled into the disposable cuvette for measuring the size and size distribution, and into the DT51070 folded capillary cell (Malvern Instruments Ltd.) for determining zeta potential. The measurements were undertaken at a scattering angle of 173° and carried out in triplicate at 25 °C.

The morphology of the internal phase of NE was examined using TEM (JEM 2011, JEOL, Tokyo, Japan). To perform the TEM observations, NE formulations were firstly diluted with distilled water in a volume ratio of 1:100 *v/v*. An amount of 10 µL of diluted sample was placed on a formvar carbon-coated copper grid (200 mesh) and then stained with 1% *w/v* aqueous solution of phosphotungstic acid. The samples were viewed under the microscope by using an accelerating voltage of 80 kV and a magnification of 10,000×.

### 3.4. Preparation of CP-NG

The optimum CP-NE formulas were selected to prepared CP-NG containing 10% CP. Modified rice powder as a gelling agent was prepared from the selected rice seeds according to the method previously described [20,22]. Rice gel base containing 30% modified rice was prepared according to the method previously described [63]. The selected CP-NE was incorporated into the gel base at room temperature until homogenous CP-NG was obtained. CP-RG was prepared by incorporating CP into the prepared gel base until a homogeneous CP-RG was obtained. The gel base was used as negative controls in the investigation of tooth bleaching efficacy and effects on enamel microhardness of the products.

### 3.5. Characterization of CP-NG

The obtained CP-NG formulations were characterized by the following tests:

#### 3.5.1. Outer Appearance and pH

The obtained CP-NG formulations were visually inspected for their outer appearance. Moreover, the color and transparency of the formulations were investigated using a colorimeter and a UV-VIS spectrophotometer. Color measurement of the colorimeter (Shenzhen Wave Optoelectronics Technology Co., Ltd., Shenzhen, China) was validated using spectrophotometer (UltraScan XE, Hunter Lab, Reston, VA, USA). An amount of 0.1 g of sample was placed on a glass slide and covered by a cover glass. The color measurement was performed on a white background. The color parameters were recorded according to the CIE L*a*b* system. Transparency of the gel was studied according to the previous method [64] with some modification. The sample was filled in a quartz cuvette and inspected by measuring % transmittance from UV-VIS spectrophotometer (UV 2450, Shimadzu, Kyoto, Japan) at 630 nm and 25 °C. Deionized water was used as a control. The pH of the formulations was determined at 25 °C using a pH meter (pH 22 Laqua Twin, Horiba). The electrode (9625-10D, 3-in-1 Electrodes, Horiba) was dipped directly into the sample, and a stable pH value was read on the display of the apparatus. Before the measurement, the apparatus was calibrated using standard buffer solutions of pH 4.0 and 7.0. The measurement was carried out in triplicate at 25 °C.

#### 3.5.2. Determination of CP Content

CP-NG formulations were analyzed for CP content using high-performance liquid chromatography (HPLC). The amount of 0.1 g of each formulation was accurately weighed and entirely dissolved in 1000 µL distilled water and subjected to a centrifuge (Sorvall ST 16R centrifuge, Thermo Fisher Scientific, Waltham, MA, USA) with a speed of 10,000 rpm for 15 min. The supernatant was filtered using a 0.22 μm filter to obtain a clear solution. This resulting solution was subjected to HPLC for analysis of CP. The data obtained were calculated using Equation (6):

(6) $$\mathrm{CP}\;\mathrm{content}\;\left(\%\right)\;=\;\left({\mathrm{CP}}_{i}/{\mathrm{CP}}_{o}\right)\;\times \;100,$$

where CP_i_ is the amount of CP in CP-NG and CP_o_ is the amount of CP original added.

#### 3.5.3. HPLC Analysis

HPLC analysis was carried out at 25 °C for assay of CP in the formulations and from the in vitro release study. A Hewlett Packard series 1100 HPLC system (Agilent Technologies, Santa Clara, CA, USA) connected with a reversed-phase column 4.6 × 250 mm (Hypersil ODS Agilent Technologies) was used as a stationary phase. The analysis performance was according to the method previously described [23] with some modification. Briefly, 1000 µL of the samples was mixed with 1000 µL of 0.1 M triphenylphosphine in a light-protected container and constantly stirred for 2 h. UV detection was at 225 nm, the injection volume was 10 μL and the flow rate was 1.0 mL/min. A mixture of acetonitrile and water was used as a mobile phase. The mobile phase was set to run in gradient, started with acetonitrile: water ratio of 50:50 (*v/v*). At 6.5 min of running time, the ratio was changed to 100:0 then at 10.0 min of running time it was changed to 50:50. This ratio was continued running until the retention time of 25 min was reached. The determination of CP was based on the oxidation of triphenylphosphine by the peroxide into triphenylphosphine oxide which can be detected by HPLC [65]. The amount of CP was determined by external quantification using triphenylphosphine oxide peak area. The calibration curve generated over CP range of 50–200 µg/mL was found to be linear with an r^2^ of 0.9997.

#### 3.5.4. Rheological Behavior and Viscosity

Rheological behavior and viscosity of the formulations was investigated using Brookfield rheometer (Rheometer R/S-CPS, plate&plate, Brookfield Engineering Laboratories, Middleboro, MA, USA) with P25 DIN plate. Shear rates from 1 s^−1^ to 120 s^−1^ for the up-curve and back from 120 s^−1^ to 1 s^−1^ for the down-curve rheograms were employed to determine the rheological properties of the formulations under shear stress. The temperature was maintained at 25 °C.

#### 3.5.5. Adhesion Study

The adhesion property of CP-NG was measured by in vitro adhesion test previously described [23] with some modification. The smooth horizontal plate with a width of 20 mm and a length of 50 mm was set next to the 30° inclined plate. The exact amount of 1 g of the test formulation was applied on the surface of the horizontal plate. A glass ball was released from the top of the inclined plane with a ball running length of 300 mm. The ball was let to further run on the horizontal plate until it stopped by adhesion force. The ball running length from the beginning of the horizontal plate to the stop point was recorded.

### 3.6. In Vitro Release and Release Kinetics Study 

In vitro drug release study was carried out using a dialysis bag with a molecular weight cut-off at 12,000 daltons (Cellu Sep^®^ T4 regenerated cellulose tubular membrane, Membrane Filtration Products, Inc., Seguin, TX, USA). The dialysis bag was activated according to the company method before starting the experiment. The accurate amount of 1 g of the test formulation was placed in the activated dialysis bag without air bubbles, and then the dialysis bag was tightly closed. Artificial saliva prepared according to the previous report [66] was used as a dissolution medium. This whole assembly was kept on a magnetic stirrer and the medium solution was stirred continuously at 100 rpm. The temperature was maintained at 37 °C. The sample was withdrawn at 5, 10, 15, 20, 30, 40, 50, 60, 120, 180, 240, 300, 360, 420, and 480 min and replaced with an equal amount of fresh dissolution medium. The withdrawal samples were subjected to HPLC analysis for the determination of the released CP. The cumulative % drug release was calculated according to Equation (7):

(7) $$\mathrm{Cumulative}\;\mathrm{drug}\;\mathrm{release}\;\left(\%\right)\;=\;\frac{V_{c}\sum_{1}^{n-1}C_{i}{+V}_{t\;}{\;C}_{n}\;}{C_{t}}\;\times \;100\%,$$

where V_c_ is the volume of media removed every time (30 mL), V_t_ is the total volume of release media (50 mL), C_i_ is the concentration of CP in the media, and C_t_ is the total CP content in CP-NG. To investigate the release kinetics of CP from the gel formulations, the data obtained from in vitro release study were examined according to several mathematical models, i.e., zero-order kinetics, first-order kinetics, Higuchi model, and Korsmeyer-Peppas model. 

### 3.7. Tooth Bleaching Efficacy and Enamel Microhardness

#### 3.7.1. Tooth Preparation

Human teeth were collected by the dentists in the Faculty of Dentistry, Chiang Mai University. This study was approved by the Human Experimentation Committee, Faculty of Dentistry, Chiang Mai University (No. 58/2016). The teeth without carries, fluorosis, or any enamel defects were selected and stored at 4 °C in saturated thymol solution until testing. For evaluation of bleaching efficacy, 50 teeth from the volunteers were randomly divided into 5 groups, each group contained 10 teeth, according to the test formulations and the controls (CP-NG1, CP-NG2, CP-RG, CP-CG, and the gel base). The whole teeth were used for this test. For evaluation of microhardness, 15 teeth from the volunteers were randomly divided into 5 groups, each group contained 3 teeth. The teeth were cut into a cubic shape of 4 mm × 4 mm × 4 mm using a diamond saw (Isomet 1000 precision saw, Buehler, Lake Bluff, IL, USA) under water cooling. Then the enamel was embedded individually in self-curing acrylic resin (Instant Tray Mix, Lang Dental Mfg. Co. Inc., Wheeling, IL, USA) in a metal ring having a diameter of 18 mm and a height of 10 mm. The enamel surface was exposed and parallel to the table of the surface.

#### 3.7.2. Investigation of Bleaching Efficacy

Before testing, the teeth were measured for baseline tooth color using a colorimeter (Shenzhen Wave Optoelectronics Technology Co., Ltd., Shenzhen, China) with color values recorded from 3 mm circular center of specimens. The colorimeter was validated for color measurement as previously described. The measurements were performed using CIE L*a*b* system. The bleaching test was carried out by following a previous protocol [67] with some modification. The teeth were individually placed over gauze moistened in artificial saliva. An exact amount of 0.1 g of each sample was placed on the tooth surface then an amount of 1 mL of artificial saliva was dropped on the sample, the tooth was kept in a close container and the temperature was maintained at 37 °C. CP-CG was used as a positive control and rice gel base was used as a negative control. After 8 h, the samples were removed, and the teeth were washed with deionized water and then measured for color changing. During the experiment, all teeth were stored at 37 °C in a closed container containing artificial saliva overnight. This bleaching process was repeated once a day for 14 days and color measurement was taken at 1, 3, 5, 7, and 14 days. The collected data were calculated to obtain the bleaching efficacy of the test formulations. The L*a*b* values were calculated for tooth color changing from baseline color by using Equation (1).

#### 3.7.3. Enamel Microhardness Test

Before the enamel specimens were exposed to the bleaching procedure, the initial enamel microhardness measurement was performed using a Vickers testing machine (STARTECH SMC-1000, Guiyang Sunproc International Trade Co., Ltd., Guiyang, China), under a vertical load of 50 g for 20 s. For each specimen, six indentations were performed on the enamel surface and the microhardness values were obtained and calculated for the mean value. The enamel surface was surrounded with the artificial saliva and then the enamel specimens were submitted to the bleaching procedure as previously described. The mean enamel microhardness values before and after the complete bleaching procedure were compared.

### 3.8. Statistical Analysis

Descriptive statistics for continuous variables were calculated and reported as a mean ± standard deviation. Data were analyzed using a one-way analysis of variance (ANOVA) and Duncan’s multiple range test (*p* < 0.05) using SPSS statistical software version 22 (SPSS Inc., Chicago, IL, USA). 

## 4. Conclusions

CP-NG formulations were prepared successfully. Characterization of the obtained developed formulations indicates that the type of carriers and drug-carrier ratios are the important parameters to achieve suitable CP-SD and CP-NE preparation. The drug-carrier ratio in CP-SD plays an important role in particle size and zeta-potential of CP-NE. The developed CP-NG formulations possess desired physical properties with a comparable buccal pH and high adhesion. The release behavior of CP from the developed CP-NG follows the first-order kinetics and the drug release mechanism is anomalous transport. The release rate of CP from CP-NG can be controlled by the drug-carrier ratio in CP-SD. This study depicts the potential of CP-NG to provide sustained drug release, while an ex vivo study substantiates the potential of the nanoemulgel formulation to enhance the bleaching effect without stimulating a reduction in enamel microhardness. It is concluded that the developed CP-NG can be served as a promising alternative product in tooth discoloration treatment.

## Figures and Tables

**Table 1:** Composition of CP-SD.

Sample	Weight Ratio (g)		
	CP	WSP	PVP
CP-SD1	1	1	-
CP-SD2	1	0.25	0.25
CP-SD3	1	0.5	0.5
CP-SD4	1	1	1

**Table 2:** Composition of CP-NE.

Sample	Weight Ratio (g)				
	CP-SD			Polysorbate 80	Water
	CP	WSP	PVP		
CP-NE1	1	0.25	0.25	0.03	1.80
CP-NE2	1	0.5	0.5	0.04	2.29
BL-NE1	-	0.25	0.25	0.03	2.80
BL-NE2	-	0.5	0.5	0.04	3.29

**Table 3:** Particle size, PdI, zeta potential, and conductivity of CP-NE.

Sample	Particle Size (nm) *	PdI *	Zeta Potential (mV) *	Conductivity (μS/cm) *
CP-NE1	262.87 ± 5.50 ^c^	0.22 ± 0.04 ^b^	−15.50 ± 1.10 ^c^	103.2 ± 9.2 ^c^
CP-NE2	409.10 ± 1.93 ^a^	0.24 ± 0.05 ^ab^	−20.73 ± 1.02 ^b^	173.6 ± 2.8 ^b^
BL-NE1	111.06 ± 5.73 ^d^	0.27 ± 0.02 ^ab^	−20.67 ± 2.16 ^b^	153.7 ± 3.4 ^d^
BL-NE2	330.63 ± 2.65 ^b^	0.29 ± 0.01 ^a^	−25.20 ± 1.50 ^a^	202.5 ± 2.1 ^a^

* Different lowercase letters indicate a significant difference (*p* < 0.05).

**Table 4:** Adhesion properties of CP-NE formulations in comparison with the commercial bleaching gel (CP-CG).

Sample	Adhesion *
	Detachment Length (cm)
CP-NE1	23.8 ± 1.0 ^a^
CP-NE2	20.9 ± 1.5 ^b^
CP-CG	2.4 ± 0.3 ^c^

* Different lowercase letters indicate a significant difference (*p* < 0.05).

**Table 5:** Color parameters and transmittance of the gel formulations.

Formulations	Color Parameters *				Transmittance (%) *
	∆L*	∆a*	∆b*	∆E*	
CP-NG1	2.86 ± 0.19 ^b^	−0.36 ± 0.03 ^b^	−0.06 ± 0.05 ^c^	2.88 ± 0.18 ^b^	30.32 ± 0.40 ^d^
CP-NG2	5.25 ± 0.10 ^a^	−0.54 ± 0.04 ^c^	−0.16 ± 0.04 ^d^	5.28 ± 0.09 ^a^	16.88 ± 0.26 ^e^
CP-RG	1.37 ± 0.09 ^c^	−0.03 ± 0.05 ^a^	0.51 ± 0.02 ^a^	1.46 ± 0.08 ^c^	85.20 ± 0.79 ^b^
CP-CG	1.50 ± 0.50 ^c^	0.08 ± 0.06 ^a^	0.13 ± 0.03 ^b^	1.51 ± 0.50 ^c^	84.59 ± 0.66 ^b^
Gel base	1.04 ± 0.21 ^c^	0.02 ± 0.03 ^a^	0.55 ± 0.05 ^a^	1.18 ± 0.19 ^c^	80.32 ± 0.40 ^c^
Control	-	-	-	-	99.91 ± 0.03 ^a^

* Different lowercase letters indicate a significant difference (*p* < 0.05).

**Table 6:** Drug content, pH, viscosity, and adhesion of the gel formulations.

Formulation	Drug Content (%)	pH *	Viscosity (Pas) *	Adhesion
				Detachment Length (cm) *
CP-NG1	99.13 ± 0.37	6.83 ± 0.01 ^b^	93.55 ± 2.50 ^b^	1.60 ± 0.18 ^b^
CP-NG2	99.32 ± 0.11	6.94 ± 0.01 ^b^	79.50 ± 1.12 ^c^	1.72 ± 0.15 ^b^
CP-RG	99.35 ± 0.34	5.88 ± 0.02 ^d^	92.60 ± 1.45 ^b^	2.00 ± 0.20 ^a^
CP-CG	99.19 ± 0.50	6.55 ± 0.01 ^c^	91.30 ± 1.35 ^b^	2.09 ± 0.20 ^a^
Gel base	-	7.20 ± 0.02 ^a^	97.69 ± 1.34 ^a^	1.50 ± 0.15 ^b^

* Different lowercase letters indicate a significant difference (*p* < 0.05).

**Table 7:** Kinetics parameters obtained from fitting the CP release experimental data to various mathematical models.

Formulation	Zero-Order		First-Order		Higuchi		Korsmeyer–Peppas		
	r^2^	k_0_	r^2^	k_1_	r^2^	k_H_	r^2^	k_KP_	n
CP-NG1	0.78	29.09	0.98	1.19	0.94	52.93	0.99	1.88	0.65
CP-NG2	0.80	16.18	0.97	0.75	0.94	44.54	0.98	1.84	0.71
CP-RG	0.79	48.17	0.98	2.50	0.96	78.56	0.98	1.94	0.52
CP-CG	0.78	30.77	0.99	1.47	0.94	58.14	0.99	1.91	0.62

## Data Availability

The data presented in this study are available upon request.

## Supplementary Materials

The following are available online at https://www.mdpi.com/1424-8247/14/2/132/s1, Figure S1: Release kinetics of CP from gel formulations according to (**a**) zero-order kinetics, (**b**) first-order kinetics, (**c**) Higuchi model, and (**d**) Korsmeyer–Peppas model.
