# Supplementary material for: Development of Controlled-Release Carbamide Peroxide Loaded Nanoemulgel for Tooth Bleaching: In Vitro and Ex Vivo Studies
Source: Pharmaceuticals (Basel). 2021 Feb 7;14(2):132. doi: 10.3390/ph14020132 (PMC7915461; doi:10.3390/ph14020132)

Supplementary Material

Development of Controlled-Release Carbamide Peroxide Loaded Nanoemulgel for Tooth Bleaching: In Vitro and Ex Vivo Studies

Siriporn Okonogi [1,2,*], Adchareeya Kaewpinta [3], Sakornrat Khongkhunthian [2,4] and Pisaisit Chaijareenont [2,5]

[1] Department of Pharmaceutical Sciences, Faculty of Pharmacy, Chiang Mai University, Chiang Mai 50200, Thailand
[2] Research Center of Pharmaceutical Nanotechnology, Chiang Mai University, Chiang Mai 50200, Thailand;); sakornratk@hotmail.com (S.K.); yodent@hotmail.com (P.C.)
[3] Interdisciplinary Program in Nanoscience and Nanotechnology, Faculty of Science, Chiang Mai University, Chiang Mai 50200, Thailand; akaewpinta@gmail.com
[4] Department of Restorative Dentistry and Periodontology, Faculty of Dentistry, Chiang Mai University, Chiang Mai 50200, Thailand
[5] Department of Prosthodontics, Faculty of Dentistry, Chiang Mai University, Chiang Mai 50200, Thailand
* Correspondence:okng2000@hotmail.com; Tel.: +66-53-944-311

Figure S1: Release kinetics of CP from gel formulations according to (**a**) zero-order kinetics, (**b**) first-order kinetics, (**c**) Higuchi model, and (**d**) Korsmeyer–Peppas model.

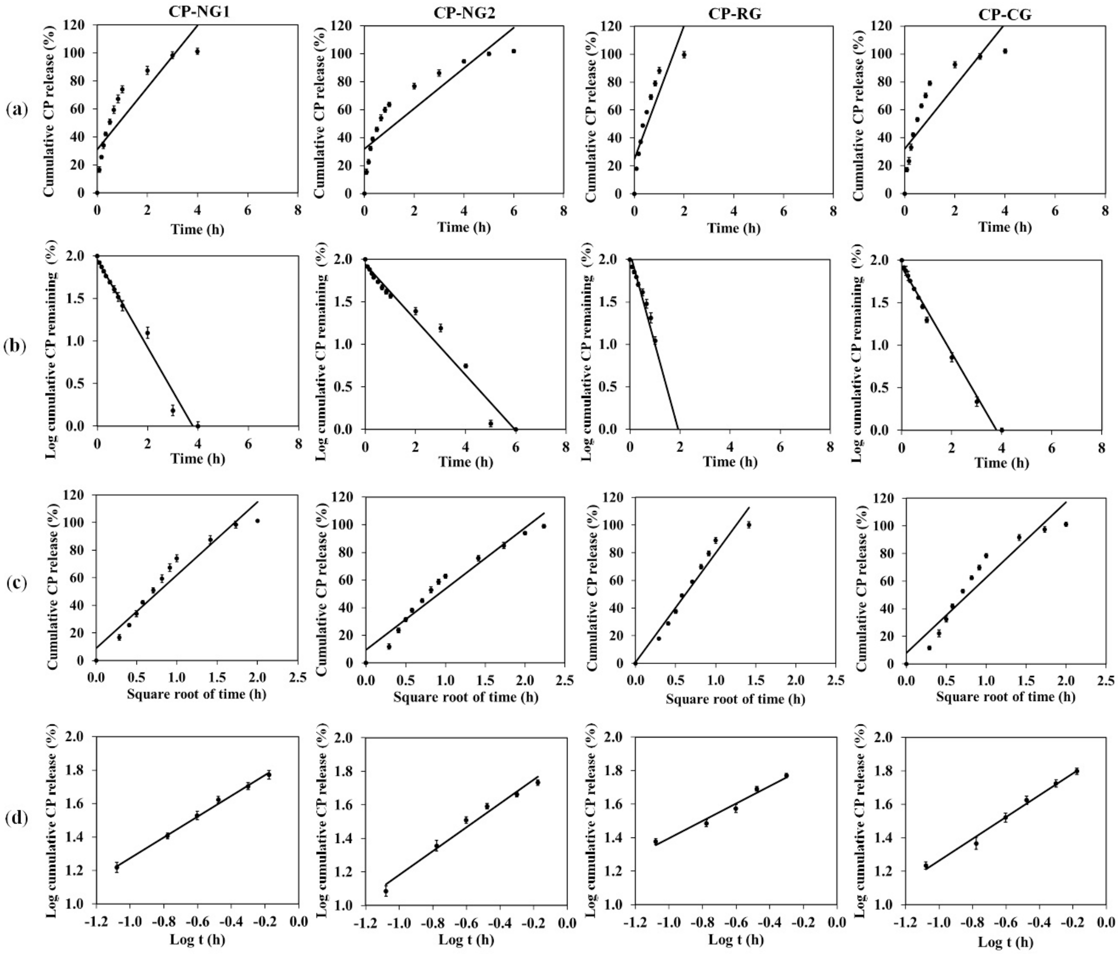

Supplement: Supplementary file 1 [file pharmaceuticals-14-00132-s001.pdf]
